# Vaccination Status and Number of Vaccine Doses Are Independently Associated with the PaO_2_/FiO_2_ Ratio on Admission in Hospitalized COVID-19 Patients

**DOI:** 10.3390/vaccines10091424

**Published:** 2022-08-29

**Authors:** Elisabetta Zinellu, Angelo Zinellu, Michela Merella, Arduino A. Mangoni, Maria Carmina Pau, Sara S. Fois, Alessandro G. Fois, Ciriaco Carru, Pietro Pirina

**Affiliations:** 1 Clinical and Interventional Pneumology, University Hospital of Sassari (AOU), 07100 Sassari, Italy; 2 Department of Biomedical Sciences, University of Sassari, 07100 Sassari, Italy; 3 Department of Medicine, Surgery and Pharmacy, University of Sassari, 07100 Sassari, Italy; 4 Discipline of Clinical Pharmacology, College of Medicine and Public Health, Flinders University, Bedford Park, SA 5042, Australia; 5 Department of Clinical Pharmacology, Flinders Medical Centre, Southern Adelaide Local Health Network, Bedford Park, SA 5042, Australia

**Keywords:** COVID-19, P/F ratio, hospitalized patients, vaccine doses received

## Abstract

Introduction: Coronavirus Disease-19 (COVID-19) vaccines reduce the risk of severe disease and mortality. However, the association between vaccination status and number of doses and the PaO_2_/FiO_2_ ratio, a clinical measure of hypoxemia associated with an increased risk of intensive care treatment and mortality, has not been investigated. Methods: We retrospectively assessed a consecutive series of 116 patients admitted to hospital with a primary diagnosis of COVID-19 between January and April 2022. Demographic, clinical, and laboratory data were collected within 24 h from admission. Results: There was a significant positive relationship between the number of vaccine doses and the PaO_2_/FiO_2_ ratio (r = 0.223, *p* = 0.012). This association remained significant after adjusting for confounders. Vaccinated patients had significantly higher PaO_2_/FiO_2_ ratios than the unvaccinated (median: 250; IQR: 195–309 vs. 200; IQR: 156–257, *p* = 0.013). Conclusion: These results highlight the importance of the number of vaccine doses received in reducing the degree of hypoxia on admission in hospitalized COVID-19 patients.

## 1. Introduction

Severe acute respiratory syndrome coronavirus 2 (SARS-CoV-2) is the causal agent of Coronavirus Disease-2019 (COVID-19) that has spread rapidly across the world and was declared as a pandemic by the World Health Organization (WHO) in March 2020 [1,2]. So far, more than 500 million cases have been confirmed with more than 6 million deaths all over the world [3]. The high contagiousness and the severity of the disease continue to have a huge impact on public health.

The clinical characteristics of SARS-CoV-2 infection vary from asymptomatic infections to severe viral pneumonia requiring oxygen administration and to more severe critical cases with acute respiratory distress syndrome (ARDS) [4,5]. A global vaccination campaign was launched in late 2020 to tackle the public health burden of the COVID-19 pandemic. COVID-19 vaccines have been shown to be effective in reducing the risk of hospitalization, admission to the intensive care unit (ICU), and mortality [6,7]. However, as COVID-19 patients continue to be hospitalized despite vaccine availability, there is ongoing research into the factors driving hospitalization, disease severity, and progress, in vaccinated patients. Respiratory symptoms and various degrees of hypoxia are common in COVID-19 patients presenting to hospital. In this context, the ratio of arterial partial pressure of oxygen (PaO_2_) to inspired (FiO_2_) partial pressure of oxygen, PaO_2_/FiO_2_, a clinical indicator of hypoxemia and respiratory failure in patients with ARDS [8,9], has also been shown to be associated with an increased risk of intensive care treatment and mortality in COVID-19 patients [10,11,12,13]. Moreover, we provided evidence of an independent association between the PaO_2_/FiO_2_ ratio on admission and prolonged hospitalization in this group [14]. Whilst national and international health authorities have emphasized the importance of receiving a full vaccination cycle, defined as three doses of COVID-19 vaccine, and current evidence suggests that a full vaccination cycle reduces the risk of severe disease and mortality, the relationship between the number of vaccine doses received and the degree of hypoxia on admission assessed with the PaO_2_/FiO_2_ has not been investigated. We sought to address this issue by investigating the associations between vaccination status, number of vaccine doses received, and PaO_2_/FiO_2_ on admission in hospitalized patients with COVID-19.

## 2. Methods

We retrospectively studied a consecutive series of 116 patients admitted with a primary diagnosis of COVID-19 to the Respiratory Disease Unit of the University Hospital of Sassari, north Sardinia (Italy), between January and April 2022. COVID-19 was confirmed by reverse transcription polymerase chain reaction (RT-PCR) in all cases. The following data were collected within 24 h of admission: parameters of comorbidity (Charlson Comorbidity Index), hypoxia (PaO_2_/FiO_2_), coagulation (D-dimer, PT, aPTT, INR, and fibrinogen) and inflammation and organ dysfunction (C-reactive protein (CRP), ferritin, procalcitonin (PCT), white blood cell count (WBC), monocytes, lymphocytes, neutrophils, platelets, mean corpuscular volume (MCV), red cell distribution width (RDW), mean platelet volume (MPV), red blood cells (RBC), hemoglobin (Hb), albumin, alanine aminotransferase (ALT), aspartate aminotransferase (AST), troponin, pro-BNP, total bilirubin, glucose, and creatinine). A brief questionnaire was administered to patients to obtain information about their vaccination status and the number of doses received. We also collected information regarding the intensity of care received, specifically in terms of respiratory support (oxygen supplementation, non-invasive respiratory support) and mortality during hospitalization. The patients were followed until in-hospital death (non-survivors) or discharge or transfer to another ward (survivors). The criteria for discharge were: (i) afebrile for at least 3 days; (ii) signs of improvement on chest CT scan or X-ray; and (iii) two consecutive negative nucleic acid tests performed at least 24 h apart. The study was conducted in accordance with the declaration of Helsinki and was approved by the ethics committee of the University Hospital (AOU) of Cagliari (PG/2020/10915).

Data are expressed as mean values (mean ± SD) or median values (median and IQR). The Kolmogorov–Smirnov test was performed to evaluate the variable distribution. Between-group differences in continuous variables were compared using unpaired Student’s *t*-test or Mann–Whitney rank sum test, as appropriate. Differences between categorical variables were evaluated by the Fisher test or chi-squared test, as appropriate. Correlations between variables were estimated using Spearman’s or Pearson’s correlation. Multiple linear regression analysis was used to assess the presence of independent associations between the PaO_2_/FiO_2_ ratio and other parameters on admission, by correcting for confounders that have a *p*-value < 0.1 in univariate analysis. Non-normally distributed variables were log10-transformed prior to analysis using parametric tests. To avoid collinearity bias, the independent association between neutrophils, lymphocytes, WBC, CRP, and procalcitonin and the PaO_2_/FiO_2_ ratio was assessed in separate models. Statistical analyses were performed using MedCalc for Windows, version 20.109-64 bit (MedCalc Software, Ostend, Belgium).

## 3. Results

The demographic, clinical, and laboratory characteristics of the study population are described in Table 1. About 26% of patients were unvaccinated whereas 8%, 22%, and 44% received one, two, and three doses, respectively. The information about the type of vaccine was not collected. However, four types of vaccines have been authorized in Italy: Pfizer-BioNTech, Moderna, AstraZeneca, and Janssen, of which the first three were the most used.

The mean values of laboratory parameters were within the normal range, except for neutrophils, CRP, procalcitonin, D-dimer, Pro-BNP, and AST (above the reference range), and lymphocytes (below range). Among the six patients transferred to ICU, five were not vaccinated (out of a total of thirty unvaccinated) and one had been vaccinated (out of a total of eighty-six vaccinated, Chi-square test *p* = 0.0025). Univariate correlation analysis showed significant negative relationships between the PaO_2_/FiO_2_ ratio and red blood cells (r = −0.251, *p* = 0.007), white blood cells (r = −0.325, *p* = 0.0004), neutrophils (r = −0.391, *p* < 0.0001), CRP (r = −0.442, *p* < 0.0001), ICU transfer (r = −0.258, *p* = 0.012), pro-BNP (r = −0.216, *p* = 0.032) and glucose (r = −0.297, *p* = 0.01) (Table 2). Significant positive relationships were observed between the PaO_2_/FiO_2_ ratio and lymphocytes (r = 0.202, *p* = 0.029), and the number of vaccines doses (r = 0.223, *p* = 0.012) (Table 2).

Multivariate regression analysis (Table 3) showed that the PaO_2_/FiO_2_ ratio was independently associated with the number of vaccine doses after adjusting for confounders that have a *p*-value < 0.1 in univariate analysis (RBC, glucose, pro-BNP, MCV, neutrophils, lymphocytes, WBC, CRP, and procalcitonin) in all the models investigated.

**Figure 1:**
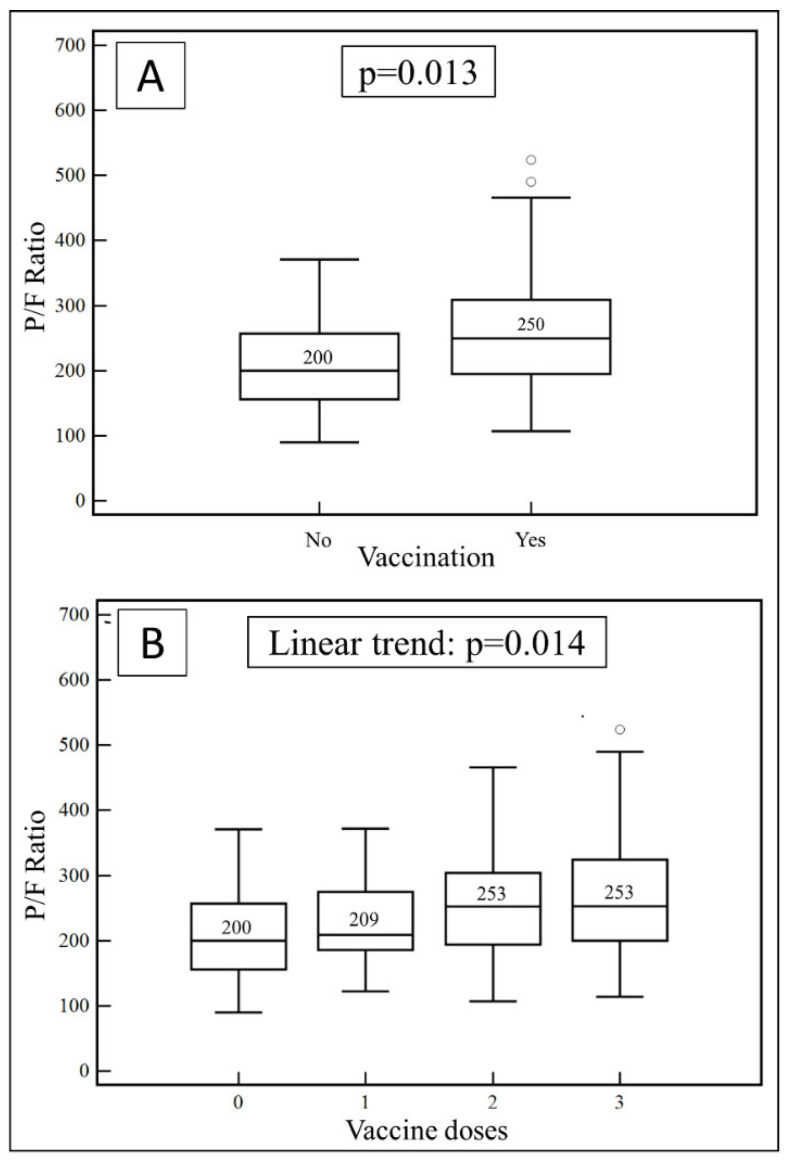
(**A**) PaO_2_/FiO_2_ ratio values on admission in non-vaccinated and vaccinated COVID-19 patients. (**B**) PaO_2_/FiO_2_ ratio values on admission on the basis of vaccine doses number administered to COVID-19 patients.

A significant difference in the PaO_2_/FiO_2_ ratio was also observed between unvaccinated patients and those receiving at least one dose of vaccine (median: 200; IQR: 156–257 vs. 250; IQR: 195–309, *p* = 0.013, Figure 1A) with a progressive and significant increase in PaO_2_/FiO_2_ values with the number of doses (no vaccine, median 200, IQR: 156–257; one dose: median 209, IQR 186–275; two doses: median 253, IQR 194–304; three doses: median 253, IQR 200–324, linear trend *p* = 0.014, Figure 1B).

**Figure 2:**
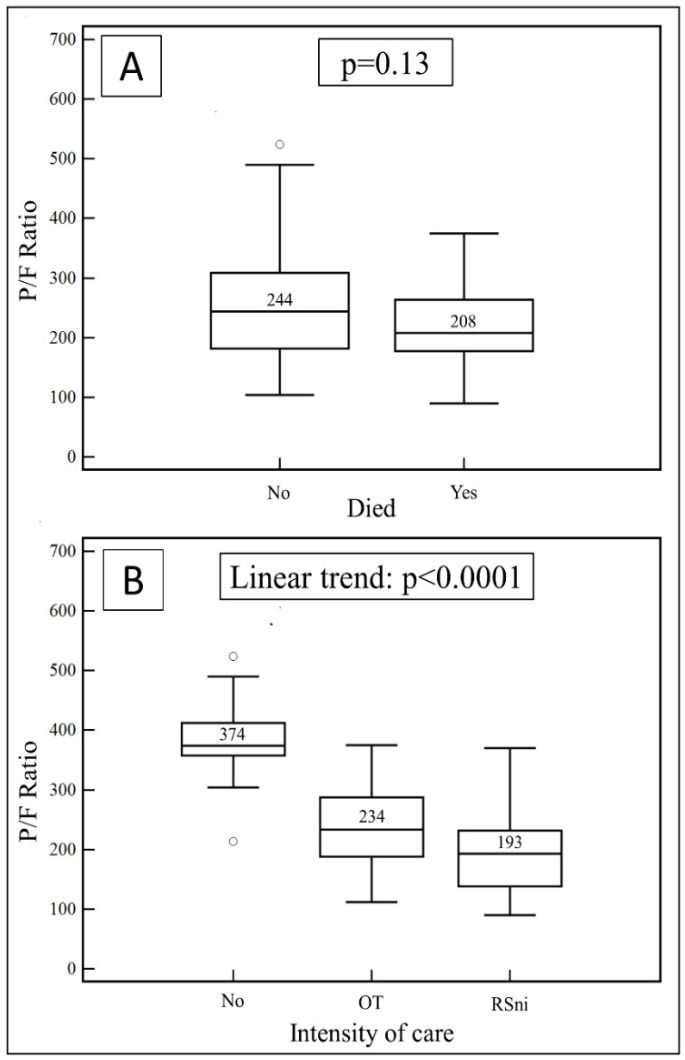
(**A**) PaO_2_/FiO_2_ ratio values on admission in COVID-19 patients sorted on whether they died or not during hospitalization. (**B**) PaO_2_/FiO_2_ ratio value on admission based on increasing intensity of care during hospitalization.

The PaO_2_/FiO_2_ ratio on admission was not significantly different between survivors and non-survivors (median: 244; IQR: 181–308 vs. 208; IQR: 177–264, *p* = 0.13; Figure 2A); however, it was significantly associated with an increasing intensity of care during hospitalization (Figure 2B).

Moreover, univariate correlation analysis showed significant negative relationships between the number of vaccine doses and BMI (r = −0.239, *p* = 0.043), intensity of care (r = −0.19, *p* = 0.04), ICU transfer (r = −0.243, *p* = 0.017), ALT (r = −0.21, *p* = 0.024), and glucose (r = −0.18, *p* = 0.048) (Table 4). Significant positive relationships were observed between the number of vaccine doses and cardiovascular disease (r = 0.185, *p* = 0.047), cancer (r = 0.247, *p* = 0.008), Charlson Comorbidity Index (r = 0.267, *p* = 0.004), and lymphocytes number (r = 0.205, *p* = 0.027) (Table 4).

## 4. Discussion

The results of our study showed, for the first time, the presence of a significant and independent positive association between COVID-19 vaccination status and, more importantly, the number of vaccine doses and the PaO_2_/FiO_2_ on admission in a consecutive series of patients hospitalized with a primary diagnosis of COVID-19. The PaO_2_/FiO_2_ ratio (also known as the Horowitz index) is defined as the ratio between the arterial oxygen partial pressure (PaO_2_) and the fractional inspired oxygen (FiO_2_) and represents a reliable measure of hypoxemia in the context of respiratory failure due to lung parenchymal damage. The PaO_2_/FiO_2_ was initially investigated as a predictor of pulmonary dysfunction in injured patients admitted to trauma services [15], before being accepted as a criterion for acute lung injury and ARDS in the American–European Consensus Conference on ARDS [16] and the Berlin definition of ARDS [9]. Severe respiratory failure represents a common complication in COVID-19 patients, and prompt recognition is of the essence. Our results confirm previously reported data regarding the association between a low PaO_2_/FiO_2_ ratio and an increase in inflammation in COVID-19 patients [17,18,19,20]. Moreover, the significant associations observed between the number of vaccine doses and the PaO_2_/FiO_2_ provide additional clinical evidence that receiving a full vaccination status, defined as three doses of the vaccine, is protective against the risk of hypoxia in patients exposed to COVID-19 presenting to the hospital. In fact, the PaO_2_/FiO_2_ has been shown to be associated with an increased risk of intensive care treatment and mortality in COVID-19 patients [10,11,12,13]. Although in our study we could not observe significant differences in the PaO_2_/FiO_2_ between survivors and non-survivors, this marker of hypoxia was significantly associated with the need for more aggressive care during hospitalization and with ICU transfer. Our results are in line with previous studies that reported that vaccination improved outcomes in hospitalized patients by reducing the risk of mortality, ICU admission, or endotracheal intubation [21]. It is likely that these results may be in part due to the higher P/F ratio value of vaccinated vs. unvaccinated patients. This is also in line with our previous observation that a higher PaO_2_/FiO_2_ ratio was independently associated with shorter hospital stay with a prognostic accuracy of 0.78 (AUC), sensitivity of 60%, and specificity of 91% [14]. Moreover, the association between the number of vaccine doses and the PaO_2_/FiO_2_ ratio are in agreement with the results of recent meta-analyses reporting that the Pfizer-BioNTech vaccine efficacy improves from 0.567, 0.837, and 0.972, respectively, after the first, second, and third dose. A similar trend was reported for Moderna that showed a vaccine efficacy of 0.72 (after first dose), 0.775 (after second dose), and 0.97 (after third dose). For AstraZeneca, the trend was similar though vaccine efficacy values were lower (0.44, 0.801, and NA) [22].

This study has some limitations due to its retrospective nature, the relatively small sample size, and the missing information regarding the type of vaccine administered, which prevented a comparison between type of vaccine and PaO_2_/FiO_2_ ratio. These issues notwithstanding, it provides useful additional support for achieving a full vaccination status in order to minimize the degree of hypoxia in case of COVID-19 exposure. Further studies are required to investigate the potential impact of the type and the timing of vaccine dose on markers of hypoxia in COVID-19 patients requiring hospital admission.

## Figures and Tables

**Table 1:** Demographic, clinical and laboratory characteristics of the studied population.

		COVID-19 Patients (*n =* 116)
Age, years		78 (66–84)
Gender (M/F)		66/50
BMI (kg/m^2^)		25.8 (23.7–30.3)
Cardiovascular disease, (no/yes)		32/84
Respiratory disease, (no/yes)		78/38
Kidney disease, (no/yes)		96/20
Diabetes, (no/yes)		92/24
Cancer, (no/yes)		100/15
Autoimmunity, (no/yes)		106/10
Charlson Comorbidity Index		1 (0–3)
P/F ratio		247 ± 90
Provenience (Emergency room/Other ward)		87/28
ICU transfer (n)		6
Deaths in the ward (n)		21
Vaccine doses, (0/1/2/3)		30/9/26/51
*Laboratory parameters*	*Reference values*	
RBC, (×10^12^ L)	*4.4–5.5*	4.45 ± 0.89
HGB, (g/dL)	*12–17.1*	12.2 ± 2.3
WBC, (×10^9^ L)	*4.8–10.8*	7.98 (5.65–10.77)
Monocytes, (×10^9^ L)	*0.16–1*	0.40 (0.30–0.60)
Lymphocytes, (×10^9^ L)	*0.9–5.2*	0.80 (0.60–1.40)
Neutrophils, (×10^9^ L)	*1.9–8*	7.14 (4.30–9.40)
Platelet, (×10^9^ L)	*130–400*	226 (161–292)
RDW, (%)	*12–14.5*	14.30 (13.10–16.45)
MCV, (fL)	*81–89*	89.4 (82.9–94.1)
MPV, (fL)	*7.2–11.1*	9.35 (8.25–10.35)
Albumin, (g/dL)	*3.3–5*	3.14 ± 0.47
Ferritin, (ng/mL)	*26–388*	397 (237–749)
CRP (mg/L)	*0–1*	7.70 (3.22–14.64)
Procalcitonin (ng/mL)	*0–0.5*	0.18 (0.07–0.63)
D-dimer, (μg/mL)	*0–0.5*	1.06 (0.68–2.27)
INR	*0.8–1.2*	1.05 (1.00–1.14)
PT, (s)	*7.5–13*	11.30 (10.80–12.15)
aPTT, (s)	*20–35*	25.65 (22.40–28.20)
Troponin, (ng/L)	*0–14*	17.15 (9.80–57.55)
Pro-BNP (pg/mL)	*0–450*	905 (274–3444)
AST, (U/L)	*5–34*	39.5 (18.0–41.0)
ALT, (U/L)	*10–55*	21.5 (15.0–35.0)
TB, (mg/dL)	*0.2–1.3*	0.77 (0.51–1.08)
Glucose, (mg/dL)	*60–99*	101 (84–132)
Creatinine, (mg/dL)	*0.72–1.25*	0.88 (0.70–1.31)

Data are presented as mean ± standard deviation or median (interquartile range). ALT: alanine aminotransferase; aPTT: activated partial thromboplastin time: AST: aspartate aminotransferase; COVID-19: Coronavirus Disease-2019; CRP: C-reactive protein; F. Female; HGB: hemoglobin; INR: international normalized ratio; M: male; MCV: Mean Corpuscular Volume; MPV: Mean Platelet Volume; P/F: PaO_2_/FiO_2;_ PT: prothrombin time; RBC: Red Blood Cells; RDW: red cell distribution width; TB: Total bilirubin; WBC: White Blood Cells.

**Table 2:** Correlation between PaO_2_/FiO_2_ ratio and demographic, clinical, and laboratory characteristics of the studied population on admission.

	Correlation Coefficient	*p*-Value
Age, years	−0.040	0.67
Gender (M/F)	−0.010	0.92
BMI (kg/m^2^)	−0.116	0.33
Cardiovascular disease, (no/yes)	0.0628	0.50
Respiratory disease, (no/yes)	0.0123	0.89
Kidney disease, (no/yes)	0.153	0.10
Diabetes, (no/yes)	0.0242	0.80
Cancer, (no/yes)	0.0607	0.52
Autoimmunity, % (no/yes)	−0.172	0.65
Charlson Comorbidity Index	0.127	0.18
Provenience (Emergency room/Other ward)	0.107	0.26
ICU transfer	−0.258	**0.012**
Deaths in the ward	−0.142	0.13
Vaccine doses, (0/1/2/3)	0.223	**0.012**
RBC, (×10^12^ L)	−0.251	**0.007**
HGB, (g/dL)	−0.0616	0.51
WBC, (×10^9^ L)	−0.325	**0.0004**
Monocytes, (×10^9^ L)	−0.0767	0.41
Lymphocytes, (×10^9^ L)	0.202	**0.029**
Neutrophils, (×10^9^ L)	−0.391	**<0.0001**
Platelet, (×10^9^ L)	−0.0711	0.44
RDW, (%)	−0.0317	0.74
MCV, (fL)	0.165	0.077
MPV, (fL)	0.0143	0.88
Albumin, (g/dL)	−0.052	0.59
Ferritin, (ng/mL)	−0.142	0.14
CRP (mg/L)	−0.442	**<0.0001**
Procalcitonin (ng/mL)	−0.172	0.077
D-dimer, (μg/mL)	−0.159	0.11
INR	0.0279	0.77
PT, (s)	0.0351	0.71
aPTT, (s)	0.133	0.15
Troponin, (ng/mL)	−0.191	0.11
Pro-BNP (ng/mL)	−0.216	**0.032**
AST, (U/L)	−0.133	0.17
ALT, (U/L)	−0.0341	0.72
TB, (mg/dL)	−0.0564	0.55
Glucose, (mg/dL)	−0.297	**0.01**
Creatinine, (mg/dL)	−0.0361	0.70

Data are presented as mean ± standard deviation or median (interquartile range). ALT: alanine aminotransferase; aPTT: activated partial thromboplastin time: AST: aspartate aminotransferase; COVID-19: Coronavirus Disease-2019; CRP: C-reactive protein; F. Female; HGB: hemoglobin; INR: international normalized ratio; M: male; MCV: Mean Corpuscular Volume; MPV: Mean Platelet Volume; P/F: PaO_2_/FiO_2_; PT: prothrombin time; RDW: red cell distribution width; TB: Total bilirubin; WBC: White Blood Cells. Numbers in bold font indicate statistical significance.

**Table 3:** Correlation between P/F ratio and demographic, clinical, and laboratory characteristics of the studied population on admission, obtained by multivariate regression analysis.

	r_partial_	*p*-Value
Vaccine doses	0.2148	**0.0377**
RBC	−0.1518	0.1443
Glucose *	−0.1446	0.1643
Pro-BNP *	0.1175	0.2595
MCV *	−0.1467	0.1583
Neutrophils *	−0.1975	0.0564

Vaccine doses	0.2151	**0.0374**
RBC	−0.2179	**0.0349**
Glucose *	−0.1966	0.0575
Pro-BNP *	0.09462	0.3644
MCV*	−0.1894	0.0675
Lymphocytes *	0.1354	0.1933

Vaccine doses	0.2376	**0.0211**
RBC	−0.1535	0.1398
Glucose *	−0.1400	0.1784
Pro-BNP *	0.1306	0.2096
MCV *	−0.1631	0.1163
WBC *	−0.1994	0.0540

Vaccine doses	0.2134	**0.0400**
RBC	−0.2821	**0.0062**
Glucose *	−0.1212	0.2471
Pro-BNP *	0.01267	0.9040
MCV *	−0.1545	0.1392
CRP *	−0.3571	**0.0004**

Vaccine doses	0.2769	**0.0086**
RBC	−0.1931	0.0699
Glucose *	−0.1566	0.1429
Pro-BNP *	0.1125	0.2937
MCV *	−0.1979	0.0631
Procalcitonin *	−0.1082	0.3128

CRP: C-reactive protein; MCV: Mean Corpuscular Volume; P/F: PaO_2_/FiO_2;_ RBC: Red Blood Cells; * Variables were log10-transformed prior to analysis. r partial: correlation coefficient of multiple linear regression analysis. Numbers in bold font indicate statistical significance.

**Table 4:** Correlations between vaccination status and demographic, clinical, and laboratory characteristics of the studied population on admission.

	Correlation Coefficient	*p*-Value
Age, years	0.170	0.07
Gender (M/F)	0.04	0.68
BMI (kg/m^2^)	−0.239	**0.043**
Cardiovascular disease, (no/yes)	0.185	**0.047**
Respiratory disease, (no/yes)	0.22	0.19
Kidney disease, (no/yes)	0.169	0.07
Diabetes, (no/yes)	0.168	0.07
Cancer, (no/yes)	0.247	**0.008**
Autoimmunity, % (no/yes)	−0.05	0.57
Charlson Comorbidity Index	0.267	0.004
Intensity of care, % (no, OT, RSni)	−0.19	**0.04**
Provenience (Emergency room/Other ward)	−0.05	0.56
Death (no/yes)	0.07	0.48
ICU transfer	−0.243	**0.017**
RBC, (×10^12^ L)	0.00	0.95
HGB, (g/dL)	−0.04	0.51
WBC, (×10^9^ L)	−0.04	0.51
Monocytes, (×10^9^ L)	0.00	0.99
Lymphocytes, (×10^9^ L)	0.205	**0.027**
Neutrophils, (×10^9^ L)	−0.12	0.06
Platelet, (×10^9^ L)	−0.02	0.70
RDW, (%)	0.04	0.55
MCV, (fL)	−0.06	0.34
MPV, (fL)	−0.08	0.20
Albumin, (g/dL)	0.00	0.98
Ferritin, (ng/mL)	−0.06	0.36
CRP (mg/L)	−0.08	0.20
Procalcitonin (ng/mL)	0.12	0.23
D-dimer, (μg/mL)	0.02	0.73
INR	0.05	0.40
PT, (s)	0.07	0.25
aPTT, (s)	0.17	0.06
Troponin, (ng/mL)	0.06	0.46
Pro-BNP (ng/mL)	0.05	0.49
AST, (U/L)	−0.16	0.10
ALT, (U/L)	−0.21	**0.024**
TB, (mg/dL)	−0.03	0.61
Glucose, (mg/dL)	−0.18	**0.048**
Creatinine, (mg/dL)	0.14	0.13

ALT: alanine aminotransferase; aPTT: activated partial thromboplastin time: AST: aspartate aminotransferase; COVID-19: Coronavirus Disease-2019; CRP: C-reactive protein; F. Female; HGB: hemoglobin; INR: international normalized ratio; M: male; MCV: Mean Corpuscular Volume; MPV: Mean Platelet Volume; OT: oxygen therapy; P/F: PaO_2_/FiO_2_; PT: prothrombin time; RDW: red cell distribution width; TB: Total bilirubin; WBC: White Blood Cells. Numbers in bold font indicate statistical significance.

## Data Availability

Data are available from the corresponding author on reasonable request.
